# Extent of malnutrition in end-stage renal disease patients

**Published:** 2014-07-01

**Authors:** Chaudhary Muhammad Junaid Nazar, John Anderson

**Affiliations:** ^1^Department of Endocrinology, University of Buckingham, Royal Gwent Hospital, NHS Trust, Wales, UK; ^2^Division of Medical Education, Postgraduate Medicine Brighton & Sussex Medical School University of Brighton, Brighton, UK

**Keywords:** End-stage renal disease, Chronic renal failure, Hemodialysis, Transplantation

**Implication for health policy/practice/research/medical education:**

Malnutrition is not specific to any stage of end-stage renal disease (ESRD), but evidently it is present even before starting renal replacement therapy (RRT). Reports published by the modification of diet in renal disease (MDRD) Study indicate that early signs of malnutrition, such as reduction in body mass index (BMI), weight and anthropometric measurements, and notable decline in urinary biochemistry parameters, including urinary creatinine excretion, was observed in chronic renal failure (CRF) patients.

The extent of malnutrition in end-stage renal disease (ESRD) patients can be discussed at different levels, including pre-dialysis, dialysis and post-dialysis, and for transplant patients.

## 1. Pre-dialysis patients

Malnutrition is not specific to any stage of ESRD, but evidently it is present even before starting renal replacement therapy (RRT). Reports published by the modification of diet in renal disease (MDRD) Study indicate that early signs of malnutrition, such as reduction in body mass index (BMI), weight and anthropometric measurements, and notable decline in urinary biochemistry parameters, including urinary creatinine excretion, was observed in chronic renal failure (CRF) patients (1). Even similar reports were found, favouring malnutrition before initiation of dialysis, indicating reductions in serum transferrin, serum cholesterol, serum IGF-1, percentage of body weight and urinary creatinine excretion as renal function deteriorated (2).

## 2. Dialysis-dependent patients

### Hemodialysis

Once patients of chronic kidney disease have been shifted from pharmacological treatment to renal replacement therapy, the extent of malnutrition becomes more severe. One research work reported serum albumin concentrations of less than 3.7 g/dl in 25% of their patient population, which included more than 12,000 hemodialysis patients (1). In the national cooperation dialysis study (NCDS), approximately 25% of patients on renal replacement therapy were found to have insufficient dietary protein and energy intake, as well as up to 40% of the patient population exhibiting levels in body fat and muscle index lower than those predicted by total-body nitrogen (TBN) (3).

### Peritoneal dialysis

Malnutrition is prevalent more in peritoneal dialysis patients than in haemodialysis patients. Some other studies also report high rates of malnutrition in continuous ambulatory peritoneal dialysis (CAPD) patients (4).

## 3. Transplant patients

The extent of malnutrition in transplant patients is still being researched and little is known about its effects. However, a few studies show that after transplantation, several parameters of patients were improved and that abnormalities in anthropometric measurements were observed in 38% of patients. One study indicates that transplant patients have some degree of depletion of visceral protein stores and decreased serum albumin concentration, specifically within the first year of transplantation. In one stable patient but with a transplant, researchers found a loss of muscle protein and also observed a reduction in muscle function (5). However, the actual prevalence of malnutrition, especially in patients with a kidney transplant, remains to be acknowledged and more research work must be encouraged to explore it further.

Several studies have encouraged patients with acute or chronic rejection to explore the importance of diet. However, the treatment and prevention of malnutrition has not been discussed about these patients. However, one can propose some intervention; especially in patients with acute rejection, the use of catabolic agents must be avoided in the early stages (6). However, in patients with chronic rejections, initiation of RRT is suggested for as early as possible, as are tapering dosages of corticosteroids.

In conclusion, the energy requirement linked with chronic uraemia is high, as is catabolic state. However, it is noted that patients who have been shifted from the pre-dialysis stage to RRT have been found to be still on a pre-dialysis diet. Patients on RRT are in need of a higher protein and calorie intake. Therefore, it is important to ensure that the dietary protein and calorie requirements of all patients are regularly checked. It is also clear that attempts should be made to encourage patients as well as their relatives to maintain adequate protein and calorie intake, especially after initiation of dialysis. Experienced dietitians must continuously check all ESRD patients’ diet in order to improve clinical outcomes. Also important is recognising early signs of malnutrition. The visit of experienced dietitians should be made compulsory for out-patients settings; and equally for hospitalisations since these patients have even lower dietary protein and calorie intakes.

If patients are so ill or malnourished that they cannot eat anything, other options for energy supplementation must be considered. These are nasogastric feeding tubes and intra-dialytic parenteral nutrition (IDPN). Only a limited number of studies evaluating the effects of enteral supplementation in malnourished CKD patients have been done. Most of these are small in capacity and show only variable degrees of success (7). It is usually a tough task discerning whether an enteral supplementation is effective and when to try more expensive and invasive procedures such as IDPN.

Recent studies suggest that IDPN acutely improves net protein synthesis and increases albumin fractional synthetic rate. Several reports have highlighted the efficient use of IDPN as a conceivable therapeutic intervention in malnourished chronic dialysis patients. In a retrospective analysis of more than 1,500 chronic hemodialysis patients treated with IDPN, it was noted that long-term use of IDPN has been found useful and helped in decreasing mortality rate. On the other hand, studies using amino acid dialysate (AAD) in peritoneal dialysis patients have offered contradictory results. In studies proposing advantages from AAD, serum transferrin and total protein concentration increased and plasma amino acid profiles tended towards one or two exchanges of AAD per day. On the other hand, increases in blood urea nitrogen (BUN) concentration associated with exacerbation of uremic symptoms as well as metabolic acidosis are potential complications of AAD (8). IDPN and AAD have been suggested as alternative methods of nutritional intervention in dialysis patients who cannot eat by the mouth. Unfortunately, studies evaluating the efficacy of nutritional procedures (IDPN and AAD) are subject to many design flaws. Therefore, their results cannot be trusted and more research work is still needed in order to evaluate efficacy, hence one should be very careful in prescribing expensive nutritional intervention.

## Authors’ contributions

CMJN completed the article. JA did critical appraisal.

## Ethical considerations

Ethical issues (including plagiarism, misconduct, data fabrication, falsification, double publication or submission, redundancy) have been completely observed by the authors.

## Conflict of interests

The authors declared no competing interests.

## Funding/Support

None.
